# Potentially Preventable Hospitalizations Among Different Subpopulations of Older Adults With Dementia

**DOI:** 10.1093/geroni/igaf122.2192

**Published:** 2025-12-31

**Authors:** Elham Mahmoudi

**Affiliations:** University of Michigan, Ann Arbor, Michigan, United States

## Abstract

Potentially preventable hospitalizations (PPHs) are expensive and harmful to people. We examined the risk of PPH among (1) everyone with dementia, (2) those in disadvantaged neighborhoods, and (4) those in rural/micropolitan areas. We used a 20% random sample of 2017-2018 Medicare claims data to select dementia patients, with at least one hospitalization (n = 67,110). Using multilevel regressions, we adjusted for age, sex, race/ethnicity, frailty index, disadvantaged neighborhood index, rurality, and number of primary care physicians and hospitals at the county. One-third of all hospitalizations were PPH, with the urinary tract infection (33.84%) being the most common. In our complete sample, PPH risk in disadvantaged neighborhoods was 18% higher than in non-disadvantaged (OR = 1.18; 95% CI: 1.08-1.30). Females were at 24% higher risk of PPH than males (OR = 1.24; 95% CI: 1.19-1.29). Black (OR = 1.15; 95% CI:1.08-1.22) and Hispanic (OR = 1.07; 95% CI: 1.00-1.16) patients had 15% and 7% higher risk of PPH than Whites, respectively. Limiting our sample to disadvantaged areas, risk of PPH for females than males increased to 35% (OR = 1.35; 95% CI: 1.12-1.62). In disadvantaged areas, there were no racial/ethnic or geographic differences in PPH risk; however, adjusted predicted PPH risk increased to 25% (95% CI:0.17-0.36) for everyone. Similarly, limiting the sample to rural/micropolitan areas, females PPH risk increased (OR = 1.30; 95% CI: 1.17-1.43) and adjusted PPH risk for disadvantaged neighborhoods increased to 27% (95% CI: 0.22-0.32). Disadvantaged and rural/micropolitan areas have substantially fewer primary care physicians. For dementia patients, practical and affordable access to primary care is one critical key to reduce PPH.

